# Silver Nanoparticle Synthesis via Photochemical Reduction with Sodium Citrate

**DOI:** 10.3390/ijms24010255

**Published:** 2022-12-23

**Authors:** Bogdan Pascu, Adina Negrea, Mihaela Ciopec, Narcis Duteanu, Petru Negrea, Lloyd A. Bumm, Oana Grad (mBuriac), Nicoleta Sorina Nemeş, Cătălina Mihalcea, Daniel Marius Duda-Seiman

**Affiliations:** 1 Renewable Energy Research Institute—ICER, Politehnica University of Timişoara, 138 Gavril Musicescu Street, 300501 Timişoara, Romania; 2 Faculty of Industrial Chemistry and Environmental Engineering, Polytechnic University of Timişoara, Victoriei Square, No. 2, 300006 Timişoara, Romania; 3 The Center for Quantum Research and Technology, Homer L. Dodge Department of Physics and Astronomy, The University of Oklahoma, 440 W Brooks St., Norman, OK 73019, USA; 4 National Institute of Materials Physics, Laboratory of Atomic Structures and Defects in Advanced Materials, Atomistilor 405A, 077125 Magurele, Romania; 5 Department of Cardiology, “Victor Babeș” University of Medicine and Pharmacy Timișoara, 2 Piața Eftimie Murgu, 300041 Timișoara, Romania

**Keywords:** silver nanoparticles, photoassisted synthesis, nanoparticle size estimation, Mie theory

## Abstract

The aim of this paper is to provide a simple and efficient photoassisted approach to synthesize silver nanoparticles, and to elucidate the role of the key factors (synthesis parameters, such as the concentration of TSC, irradiation time, and UV intensity) that play a major role in the photochemical synthesis of silver nanoparticles using TSC, both as a reducing and stabilizing agent. Concomitantly, we aim to provide an easy way to evaluate the particle size based on Mie theory. One of the key advantages of this method is that the synthesis can be “activated” whenever or wherever silver nanoparticles are needed, by premixing the reactants and irradiating the final solution with UV radiation. UV irradiance was determined by using Keitz’s theory. This argument has been verified by premixing the reagents and deposited them in an enclosed space (away from sunlight) at 25 °C, then checking them for three days. Nothing happened, unless the sample was directly irradiated by UV light. Further, obtained materials were monitored for 390 days and characterized using scanning electron microscopy, UV-VIS, and transmission electron microscopy.

## 1. Introduction

Recently, metallic nanoparticles have aroused a special interest due to their physical and chemical properties, which are superior to the bulk material, as they can be modified depending on the purpose of the final material. Among such nanoparticles, AgNPs became of interest for their applicability in a variety of functions, such as antimicrobial [1,2]; water treatment [3]; contrast agents for magnetic resonance imaging, MRI imaging or computer tomography, CT [4]; catalysts [5]; biosensors [6]; SERS applications (surface enhanced Raman spectroscopy); in photovoltaic cells (three times more charge generation) [7,8], etc.

Generally, the properties of silver nanoparticles strongly depend on their morphology, e.g., AgNPs with small dimensions and spherical or quasi-spherical geometric shape release a larger amount of Ag^+^ due to their larger specific surface area [9]. Therefore, silver ions can react with the –SH groups of enzymes and proteins which are bound by the cell wall of the bacteria [10], and even more so, Ag^+^ ions released from the AgNPs can penetrate the bacterial cell wall, reaching the cytoplasm and thus degrading the chromosomal DNA [10,11]. Silver nanoprisms are suitable for SERS applications (the SERS intensity using silver nanoprisms of rhodamine 6G was 20× stronger than that of the silver nanospheres [12], photovoltaic cells (generated three times more charge) [7], DNA detectors [13], and Hg^2+^ sensors [14]. These applications depended not only on the adjustable intensity of the plasmonic band of the nanoprisms, but also on their small radius of curvature. On the other hand, nanoprism morphology leads to a huge enhancement of the electric field around the tips of the triangular nano-prisms [15], which makes them very useful in the aforementioned applications.

Silver nanofibers, due to their excellent optical and electrical properties [16], are very effective in applications such as OLEDs [17], relative humidity sensors, ammonia sensors [18], etc.

To date, several methods have been used to synthesize silver nanoparticles: chemical reduction [19], laser ablation [20,21,22], photochemical synthesis [23], “green” syntheses using plant extracts [19,24,25], evaporation–condensation [26], micro-emulsion [27], etc.

Of all these methods, photochemical synthesis is simple, versatile, and efficient, has a high spatial resolution [23], and does not require the addition of a chemical reducing agent. Moreover, using the photochemical synthesis, one can obtain nanoparticles in various media, such as glass [23], zeolite crystals [28], polymeric films [29], etc. The photochemical synthesis of silver nanoparticles has some advantages, including that the synthesis can be performed at room temperature [30], and that the equipment used is simple, inexpensive, and controllable. However, even with all of these advantages, the photochemical approach is not commonly used. 

The use of a stabilizing agent is important in order to control the homogeneous formation of nanoparticles with the desired size and geometric shape [31,32], but also to prevent agglomeration and possible precipitation of the nanoparticles [32,33]. A multitude of reagents can be used as stabilizers, starting from tri-sodium citrate (TSC, Na_3_C_6_H_5_O_7_) [34,35], chitosan [36], poly-vinyl-pyrrolidone (PVP) [37], EDTA [38], etc.

A disadvantage of TSC is that it is also a weak reducing agent, in addition to its role as a complexing agent, thus making it difficult to obtain a monodisperse synthesis. However, the photochemical synthesis of nanoparticles using TSC as a capping agent remains popular, because it forms relatively stable complexes [39], and implicitly, one can obtain a stable solution. 

**Scheme 1:**
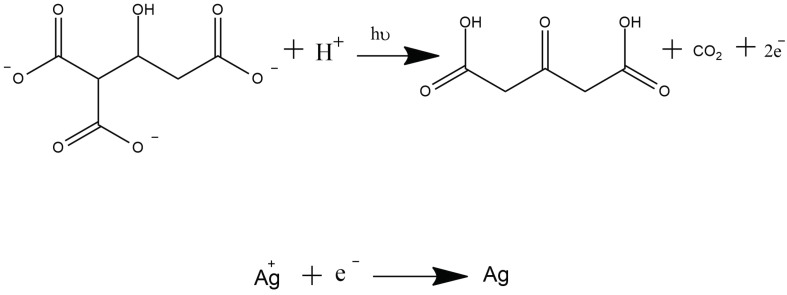
Silver reduction mechanism.

Thus, the reaction mechanism involving this synthesis can be as follows (Scheme 1) [40]:

Firstly, the decomposition of trisodium citrate by the UV irradiation occurs to form acetone-1,3-dicarboxylate.

**Scheme 2:**
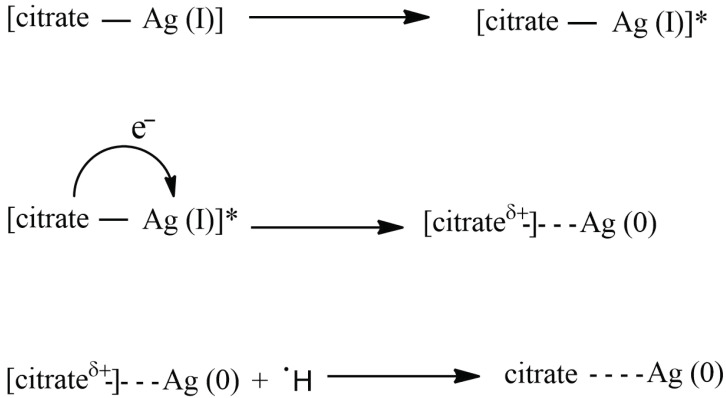
Mechanism of silver reduction by citrate.

The second process is a complexing and excitation process (Scheme 2), as proposed by Shengchun Yang et al. [41]:

The photochemical synthesis can be used to obtain AgNPs with different morphologies using irradiation sources with certain wavelengths [42]. For example, using a light emitting diode (LED), which irradiates at a λ of 467 nm (specific to the colour of blue), one can obtain silver nanoparticles with a majority of their morphology in the shape of a decahedron, while at a λ of 630 nm (specific to the colour of red), one can obtain silver nanoprisms [43].

There are many studies which present different approaches to photochemical synthesis of silver nanoparticles, such as using plant extracts [44], chitosan [29], TSC [34]. Most of the TSC syntheses carried out until now have offered some explanations regarding the optimization of the synthesis [45,46], but the optimization of this type of synthesis, which uses only photons to “activate” TSC reducing capabilities, without any additional reagent (as a reducing or stabilizing agent), is still an issue to be addressed. Thus, one of the main aims of this paper is to optimize the photochemical synthesis of AgNPs by adjusting different parameters, such as TSC concentration, UV intensity, UV exposure time, and the monitoring of the resulting solution.

Thus, this paper presents a photochemical method for the synthesis of AgNPs, using AgNO_3_ as a precursor for AgNPs and UV radiation as an “activator” for TSC, in order to begin the reduction, with TSC as the stabilizing agent. The size of the AgNPs were estimated using Mie theory. The stability of the AgNPs was studied over 390 days (13 months).

## 2. Results and Discussion

In this section of the article, we attempt to optimize the synthesis of silver nanoparticles by studying different synthesis parameters, such as TSC concentration, UV irradiation time, and UV intensity (irradiance calculation using Keitz’s theory). Thus, after optimizing the synthesis parameters, we continue with the electron microscopy characterization of the material (SEM/EDX, TEM), Mie theory characterization of the obtained silver nanospheres, and stability studies, which consist of periodic measurement of the optimal silver nanoparticle solution using UV-VIS spectroscopy.

### 2.1. Synthesis Optimization

#### 2.1.1. TSC Concentration

**Figure 1:**
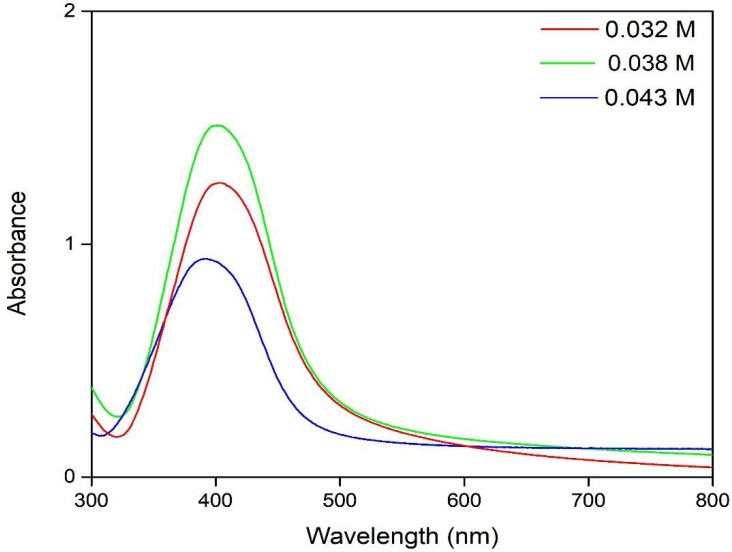
The influence of the concentration of TSC on AgNP synthesis.

We explore the influence of the TSC concentration on AgNP synthesis. Three TSC concentrations (0.032 M; 0.038 M; 0.043 M) were used. The distance (3.5 cm) from the UV lamp and the irradiation time (60 min) were kept constant. The resultant AgNPs were evaluated from their extinction spectra (Figure 1).

The localized plasmon resonance (LSPR) at ~400 nm indicates that the predominant morphology of the silver nanoparticles is spherical [47,48,49].

We conclude that the 0.038 M TSC concentration is the optimal concentration because the absorbance is the highest for that preparation, indicating that it produced the highest concentration of nanoparticles [50,51].

In the case of 0.043 M, a precipitate was observed (possibly silver), which can explain the lower absorbance in comparison to the other two samples. Thus, the subsequent research was continued with a TSC concentration of 0.038 M.

#### 2.1.2. UV Irradiation Time

**Figure 2:**
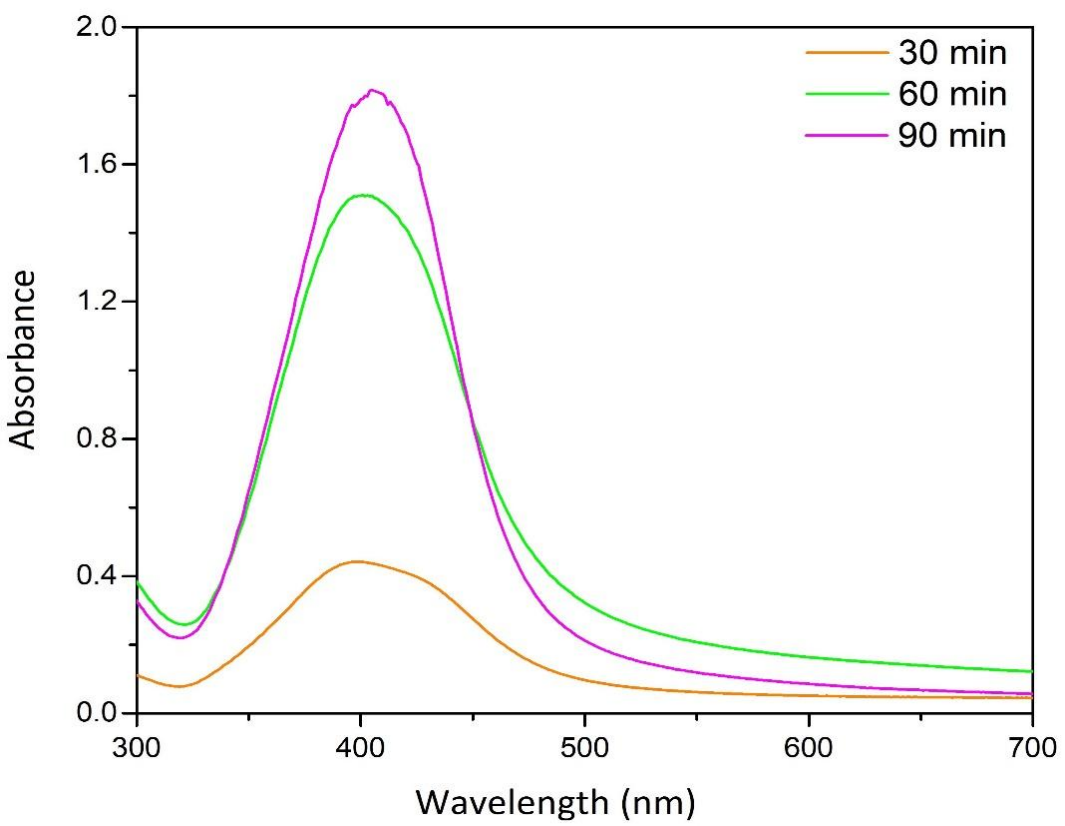
The effect of UV irradiation time on AgNP synthesis.

To determine the effect of the irradiation time, the TSC concentration (0.038 M), and the source–sample distance (3.5 cm) were kept constant, varying only the irradiation time. The resultant AgNPs were, again, evaluated from their extinction spectra (Figure 2).

The sample which was irradiated for 90 min exhibited the highest absorbance. However, the extinction maximum was red shifted, indicating a presence of some coarsened nanoparticles [52,53], as well as some aggregated nanoparticles [54]. The sample which was irradiated for 30 min exhibited the lowest absorbance, indicating the lowest concentration of AgNPs [50,51]. The sample which was irradiated for 60 min excited an absorbance close the that of the 90 min sample, but did not show coarsening or aggregation. Therefore, 60 min was selected as the preferred irradiation time for our studies.

#### 2.1.3. UV Intensity

**Figure 3:**
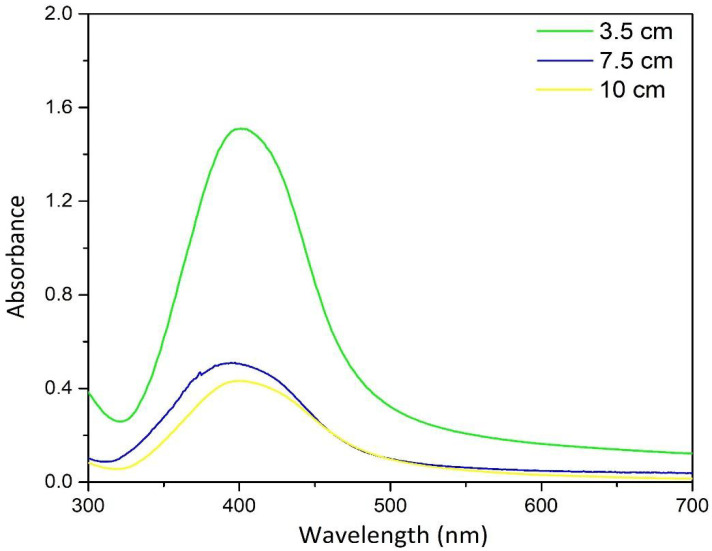
The effect of the distance between the UV irradiation source and sample on AgNP synthesis.

To determine the effect of the UV intensity on AgNP synthesis, the source–sample distance was varied (3.5 cm, 7 cm, 10 cm) while the TSC concentration (0.045 M) and the irradiation time (60 min) were kept constant. The resultant AgNPs were, again, evaluated from their extinction spectra (Figure 3).

From these extinction spectra, we concluded that the distance of 3.5 cm from the irradiation source provided the optimal UV intensity, because the concentration of AgNPs (relatively high absorbance) was much higher compared to the other two samples (7 and 10 cm, respectively). 

Thus, in order to determine the necessary UV intensity to obtain an optimal synthesis, the irradiance (I) can be calculated using Keitz’s formula [34,55,56,57]:

$$P=\frac{I2\pi^{2}\mathrm{DL}}{2\alpha +\sin2\alpha }$$

From here, we can obtain the intensity “I”:

$$I=\frac{P\;\left(2\alpha +\sin2\alpha \right)}{2\pi \mathrm{DL}}$$

where:

P—UV lamp power [W]

I—radiation intensity [W m^−2^]

L—UV tube length [m]

D—distance [m]

α = arctan $\left[\frac{L}{2D}\right]$ rad; sin α = $\frac{L}{\sqrt{4D^{2}+L^{2}}}$; cos α = $\frac{2D}{\sqrt{4D^{2}+L^{2}}}$; sin2 α = 2 sin α cos α.

Following the calculations performed for the three distances considered for the synthesis, the results are presented in Table 1. 

From the obtained results, one can notice a correlation between the UV-VIS spectra (Figure 2) and the obtained irradiance.

After the conversion of distance into intensity, the dosage was calculated in order to determine the optimal UV radiation dosage. For this calculus, we used the optimal irradiance (214 W/m^2^) and the studied irradiation time, thus resulting in the following (Table 2):

From the experimental UV-VIS results, a dosage of 770,400 J/m^2^, which is equivalent to the distance of 3.5 cm (the optimal distance, with an irradiance of 214 W/m^2^), and 60 min of time represents the optimal dosage for this synthesis.

#### 2.1.4. Summary

In conclusion, in order to obtain the optimal synthesis of AgNPs, the concentration of 0.029 mol AgNO_3_/L and 0.038 mol TSC/L, with an irradiation time of 60 min and a distance between the sample and UV of 3.5 cm, should be used. The sample which was synthesized with the optimal parameters was further studied. 

### 2.2. Scanning Electronic Microscopy and Energy Dispersive X-ray Analysis 

**Figure 4:**
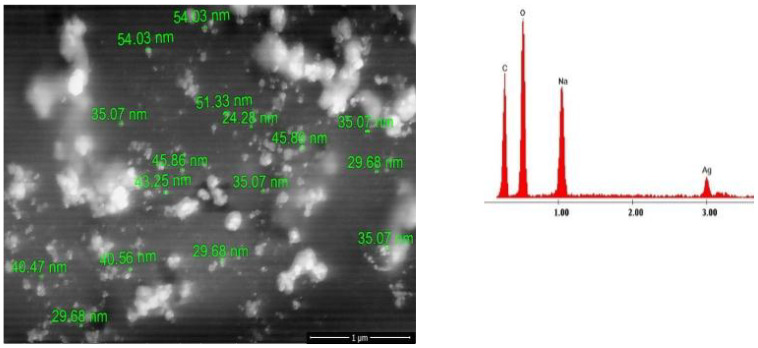
SEM and EDX analysis for the AgNPs.

The morphology of the AgNPs was measured using SEM, and qualitative elemental analysis was performed using EDX (Figure 4).

SEM was performed as a preliminary analysis to evaluate the morphology of the AgNPs, and to determine a preliminary particle size. From the analysis, we can conclude that the measured nanoparticles are of spherical shape, with diameters measuring ~40 nm, and that they are quite polydisperse. EDX analysis confirms the presence of Ag. Na, C, and O are specific for TSC. The SEM and EDX analyses were performed a few hours after synthesis of the AgNPs.

### 2.3. Transmission Electron Microscopy

**Figure 5:**
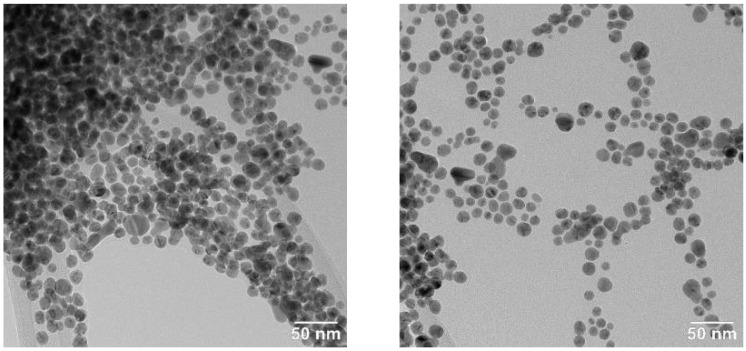
TEM images from different areas of the sample, showing spherical and quasi-spherical AgNPs.

**Figure 6:**
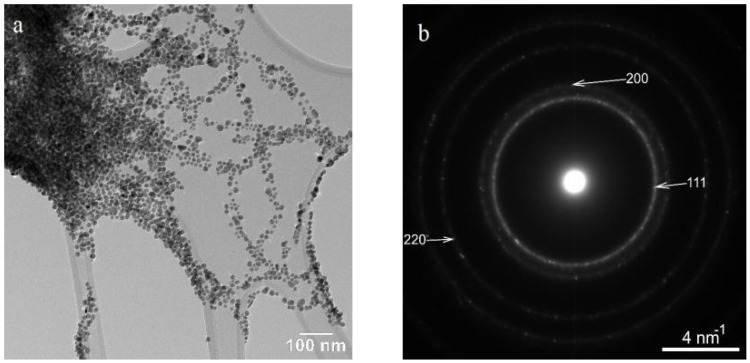
An agglomeration of AgNPs (**a**) and the electron diffraction pattern revealing the FCC (face-centered cubic) structure of Ag (**b**); the most intense diffraction rings correspond to (111), (200), and (220) crystallographic planes.

**Figure 7:**
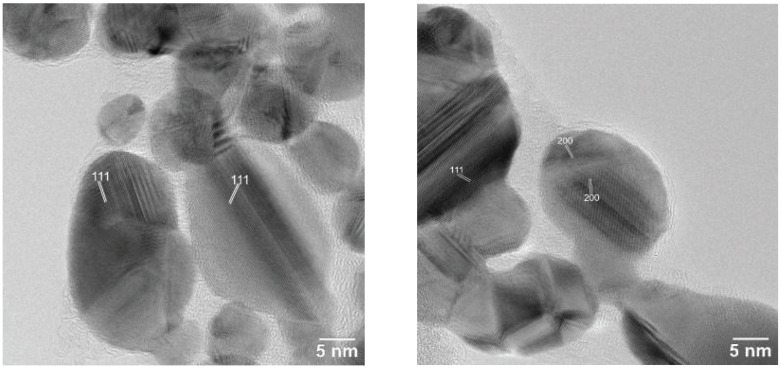
HRTEM images of AgNPs; the lattice fringes correspond to (111) and (200) crystallographic planes of FCC (face-centered cubic) AgNPs.

**Figure 8:**
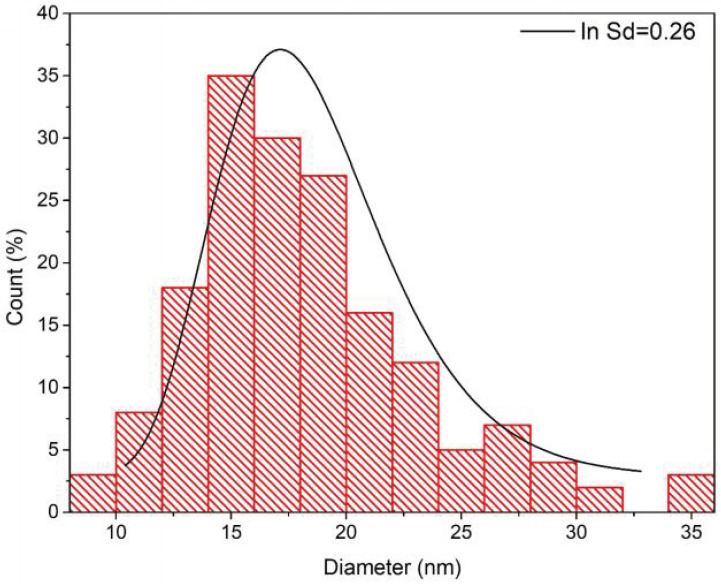
Size distribution based on TEM analysis.

The TEM analysis was performed in order to obtain a better evaluation of the nanoparticle size (the maximum, minimum, and distribution, whilst the SEM analysis was preliminary, conducted in order to obtain an idea of the nanoparticles’ shapes and sizes. In Figure 5, Figure 6 and Figure 7, the TEM micrographs are presented for the optimal sample (0.029 M AgNO_3_, 0.038 M TSC, irradiation time of 60 min, and a distance from the radiation source of 3.5 cm). The particle size distribution obtained based on the TEM images it is depicted in Figure 8.

From the obtained results, we can conclude that the nanoparticle sizes are slightly polydisperse (which can also be seen from the UV-VIS spectra analysis), with a majority of them being around 15 nm. Even though a majority of them are around 15 nm, there are still some coarse ones present, which are ~35 nm.

### 2.4. Stability Study

In order to perform this study, the obtained sample was kept in the dark, at room temperature (~25 °C), and wrapped in aluminum foil in order to prevent the interference of ambient light.

**Figure 9:**
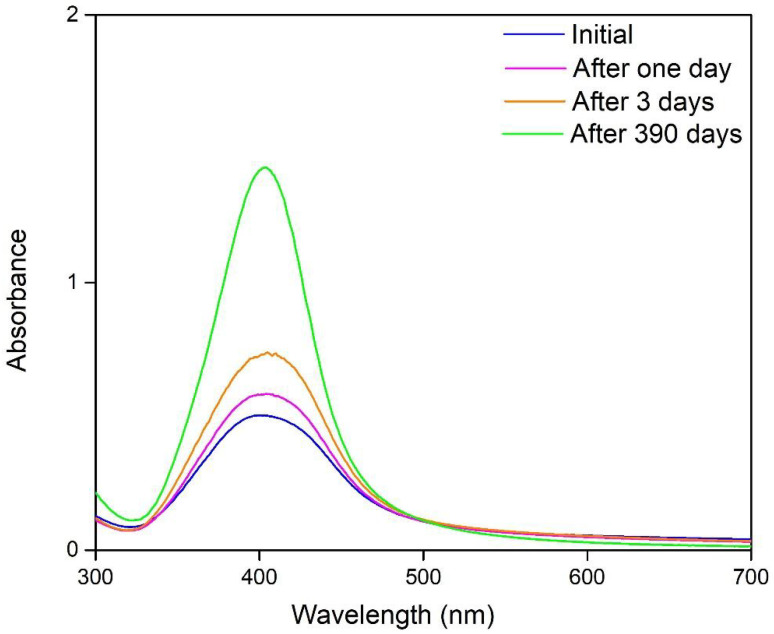
Stability studies.

The stability of the sample was studied by performing UV-VIS spectra over time. The results are shown in Figure 9.

From the spectra, it can be observed that the absorbance increases continuously, and the full width at half maxima decreases with the increase in absorbance (the initial spectra has a FWHM of 85 nm (Gaussian). After 390 days, its full width at half maxima is 66 nm (Gaussian)), thus indicating the fact that the nanoparticles are more monodispersed [58,59]. Their diameter is also smaller compared to the initial one [35,60,61], implicitly increasing the concentration of nanoparticles as their diameter decreases. This issue can be explained by the fact that TSC has been activated by the UV irradiation [40]. Thus, this type of synthesis has the benefit of a controlled “activation” in order to obtain AgNPs, but the disadvantage is that the reduction process cannot be stopped instantaneously; the citrate continues reducing continuously until the reaction is completed. In conclusion, in order prevent this from happening, we suggest the use of an additional stabilizing (besides TSC) agent to form a more stable complex.

The solution with the obtained silver nanoparticles was monitored for 390 days, revealing changes in the UV-VIS spectra during the study period.

### 2.5. Absorption and Scattering by a Sphere; Mie Theory Size Assessment

**Figure 10:**
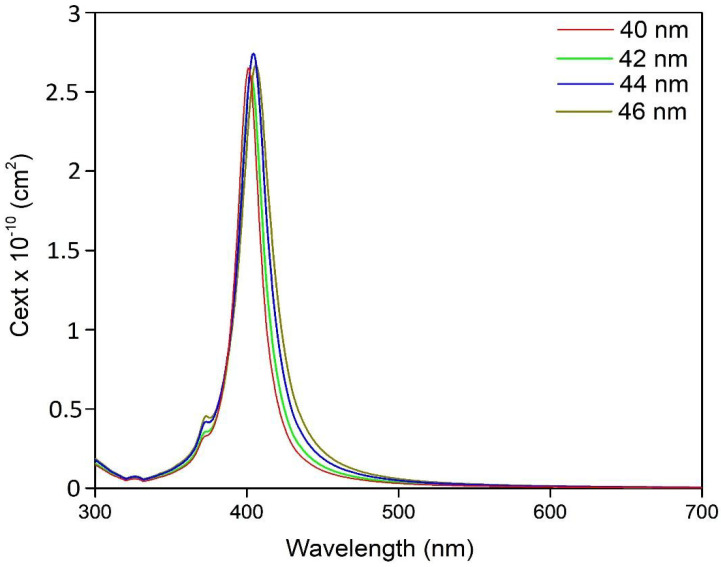
Extinction cross-section for AgNPs with a diameter of 40–46 nm.

In order to estimate the size of the nanoparticles, a first step involves obtaining the theoretical spectra “*C_ext_/C_sca_/C_abs_* vs. *Wavelength*” for the diameter of nanoparticles, which presents the localized surface plasmon resonance at a similar wavelength to the experimental one, depicted in Figure 10 (the wavelength is closely related to the morphology of the nanoparticles) [35,62,63,64].

The spectra represented in this figure were obtained using the Mie Plot software [65], and they are representative of silver nanospheres with a diameter of 40–46 nm. In order to be able to compare the theoretical results with the experimental ones, it is necessary to perform the following calculations.

Thus, from the theoretical results, we will use the values obtained for C_ext_, to calculate the molar absorption coefficient, ϵ, for each spectrum [66,67]. 

Therefore: ϵ = N_A_ C ln10 (10^−3^ $\frac{L}{{\mathrm{cm}}^{3}}$) where: N_A_ = 6.0221408 × 10^23^ (mole^−1^) 

C = extinction cross section (cm^2^)

The obtained result can be used, together with the molar absorption coefficient, ϵ, to determine the absorbance of each spectrum using the Beer–Lambert law [68]:

A = ϵ c l

where: A = Absorbance

ϵ = molar absorption coefficient (M^−1^ cm^−1^)

c = the molar concentration of the solution (M)

l = path length (cm)

**Figure 11:**
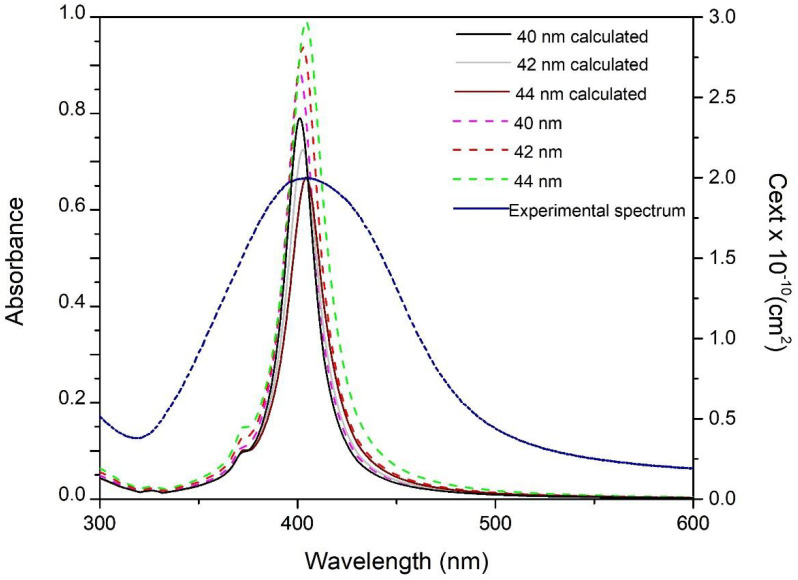
Theoretical obtained spectra for AgNPs with diameters from 40–46 nm, and the experimentally obtained spectrum.

Applying this analysis, we obtained the following results, presented in Figure 11.

The dark-colored lines (light grey, black, and brown) are the theoretical calculated spectra, while the dotted ones represent the extinction cross-section spectrum.

To obtain a more precise comparison, the sample of AgNPs was diluted in order to achieve an absorbance of <1. Following the obtained results, it is evident that the absorbance of the theoretically calculated spectra decreases with the growth of the nanoparticles diameter, thus indicating the fact that the nanoparticle concentration decreases (lower absorbance), with the increasing size of the particles. It can also be observed that the wavelength at which the LSPR occurs (401 nm for D = 40 nm; 403 nm for D = 42 nm; 404 nm for D = 44 nm) is directly proportional to the increasing size of AgNPs, indicating a correlation between AgNP morphology and the LSPR wavelength, a fact which has been demonstrated in several papers [59,60,62,69]. 

Comparing the spectra between them, it results that the LSPR of the synthesized material occurs at a wavelength of 404 nm, and the theoretically obtained spectrum (the one which “overlaps” with the experimental one) has an LSPR at a wavelength of 406 nm. Therefore, we conclude that in the experimental solution, the diameter of the obtained nanoparticles is approximately ~44 ± 3 nm. It can also be seen that the synthesized material has a 99 nm FWHM with a regression coefficient of R^2^ = 0.99643 (Gaussian fit), a fact which indicates that the obtained nanoparticles are polydisperse [58,59,70].

Judging from the calculated spectrum and the experimental one, we can conclude that a majority of the nanoparticles present in the solution should be around ~44 nm.

Of course, it would be ideal for all AgNPs to have the same diameter (monodisperse), but this is often not possible (depending on the used reducing agent [71]). In order to obtain results as close as possible to the real values, the following steps should are necessary: (i) the rapid reduction in AgNO_3_ will lead to acquiring a monodisperse solution [72,73]; (ii) ultracentrifugation or ultrafiltration in order to remove the coarse colloids as well as the colloidal aggregates from the resulting solution; and (iii) the determination of the silver concentration via AAS (atomic absorption) or ICP-MS (inductively coupled plasma mass spectrometry) depending on the concentration of the silver in the solution, after the ultracentrifugation or ultrafiltration step, to determine the exact concentration of silver. 

**Figure 12:**
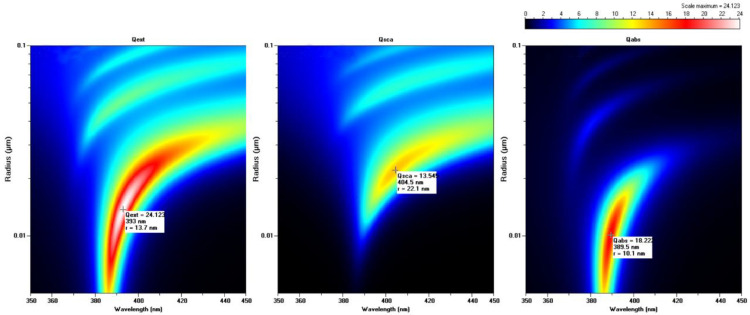
Qext, Qsca, and Qabs as a function of particle radius r and wavelength λ.

In addition, the theoretical extinction efficiency Q_ext_, scattering efficiency Q_sca_, and absorption efficiency Q_abs_ [74] for the silver nanospheres with radii from 5–100 nm (10–200 nm diameter) are presented in Figure 12.

The color indicates the magnitude of Qext, and, implicitly, the maximum values depending on the nanoparticle radius. Q_ext_ is the sum of Q_sca_ and Q_abs_ [75].

The size differences between SEM, TEM, and Mie theory could be related to the fact that the obtained nanoparticles are polydisperse (which can be seen from UV-VIS spectra, SEM, and TEM), and that the reaction did not complete during the irradiation period. The coarse nanoparticles and the aggregates could be removed from the solution by subjecting the solution to an ultracentrifugation and/or ultrafiltration process, in order to obtain a more precise Mie theory size assessment.

## 3. Materials and Methods

For the synthesis of silver nanoparticles, the following were used: AgNO_3_ (p.a. Merck) as a precursor for AgNPs, trisodium citrate dihydrate (TSC) (ACS reagent, >99% purity) as a stabilizer and as a reducer, and an UV lamp (Tungsram 18 W, having an UV output of 6 W, with λ of 254 nm) [40]. 

**Figure 13:**
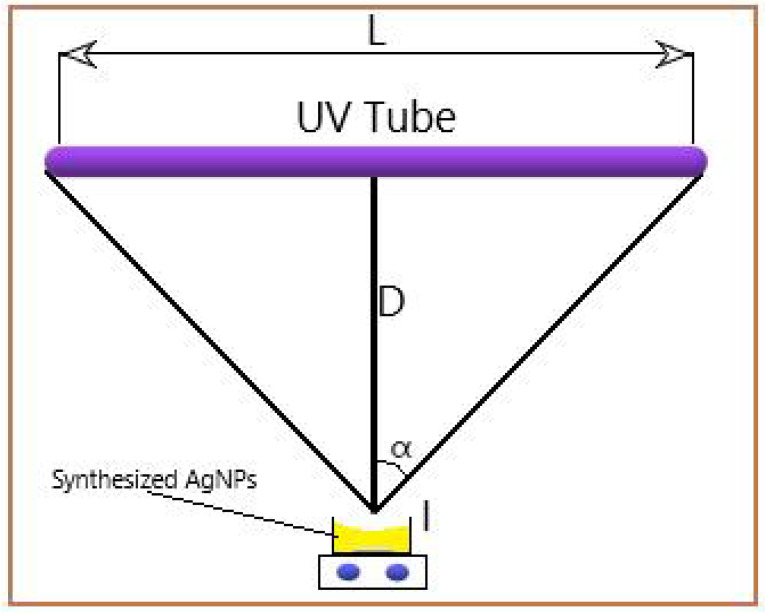
Schematic representation of the synthesis.

To obtain information regarding the synthesis process, the following parameters were varied: (i) TSC concentration (0.032 M; 0.038 M; 0.043 M), (ii) UV irradiation time (30 min, 60 min, and 90 min), (iii) the distance between the irradiation source and the sample (3.5 cm, 7 cm, and 10 cm), (iv) stability studies and evolution over time. All solutions were prepared using double distilled water. The synthesis of AgNPs was carried out as follows. To 30 mL of one of the TSC solutions, a volume of 1 mL of AgNO_3_ (0.029 M) was added dropwise with constant stirring. The resulting solution was left to be stirred for 10 min. The next step was the transfer of the solution to a Petri dish (to facilitate the penetration of the UV radiation through the entire volume of the solution). The Petri dish with the solution was subjected to UV irradiation, while keeping the solution under magnetic stirring. It is worth mentioning that the Petri dish was in the middle of the UV lamp, the UV lamp was oriented above the Petri dish, and both were enclosed in a box without any interference from outside. The schematic representation of the synthesis of AgNPs is presented in Figure 13 [55].

The tube length (L) was 40 cm, and the distance traveled by the radiation (D) was 3.5 cm, 7 cm, and 10 cm, respectively, to the irradiated sample (I). The UV output of the tube was given by the manufacturer, and equaled 6 W.

The obtained sample was kept in the dark, at room temperature (~25 °C), and wrapped in aluminum foil in order to prevent the interference of ambient light. The resulting AgNP solutions were characterized as follows. 

UV-vis spectra were obtained by using a Varian Cary 50 spectrophotometer. Nanoparticle morphology and composition was analyzed using scanning electron microscope (SEM) Quanta Feg 250, which was equipped with energy dispersive x-ray analysis (EDX). A more detailed analysis employed transmission electron microscopy (TEM) using the JEOL JEM-2100 with selected area electron diffraction (SAED). Long-term stability was studied via UV-VIS spectroscopy.

## 4. Conclusions

This study demonstrates a simple method for photoassisted synthesis of AgNPs. In this method, we used UV radiation to “activate” the reducing capabilities of TSC (unlike the classical Turkevich method, in which the solution needed to be heated in order to synthesize nanoparticles).

Additionally, the irradiance was determined, and it was observed that there exists a close correlation between the calculated irradiance and the experimental obtained extinction spectra in Section 2.3. The difference between the absorbances of the 7 cm and 10 cm spectra are not significant, and the same can be said about the calculated irradiances of the same distances. This indicates the close correlation between the irradiance and the resulting extinction maxima. 

A simple and efficient theoretical determination of nanoparticle size was conducted and explained, using the theory developed by Gustav Mie, by comparing the theoretical obtained spectra and the experimental one, as well as the confirmation of the results using the SEM analysis and TEM analysis. 

The stability of the obtained silver nanoparticles was studied for 390 days, and they showed good stability overall. Even though some modifications still occurred in the solution, no precipitate was observed.

One of the major advantages that this type of synthesis offers is the benefit of a controlled “activation.” Thus, the reactants may be premixed, kept in the dark, and exposed to UV irradiation when one wishes to obtain AgNPs.

## Figures and Tables

**Table 1:** Irradiance results.

Distances between the UV Lamp and Sample [cm]	Irradiance [W/m^2^]
3.5	214
7	106
10	82

**Table 2:** Radiation dosages.

Irradiation Time [s]	Irradiance [W/m^2^]	Dosage [J/m^2^]
1800	214	385,200
3600	214	770,400
5400	214	1,155,600
